# Design-Space Exploration of Sensing Margin in 1T-nC Ferroelectric Random-Access Memory Considering Capacitor Length and Electrode Work Function Variations

**DOI:** 10.3390/mi17101124

**Published:** 2026-09-27

**Authors:** Jehyeok Jung, Munhyeon Kim, Sihyun Kim

**Affiliations:** 1 Department of Electronic Engineering, Sogang University, Seoul 04107, Republic of Korea; ryan0313@u.sogang.ac.kr; 2 Department of Electrical and Information Engineering, Seoul National University of Science and Technology, Seoul 01811, Republic of Korea

**Keywords:** FeRAM, process variation, sensing margin, wet etch, work function, high-density memory, non-volatile memory, TCAD simulation

## Abstract

The rapid advancement of artificial intelligence (AI) necessitates high-performance computing architecture. While compute express link (CXL) technologies facilitate memory expansion, conventional dynamic random-access memory (DRAM) encounters fundamental limitations in power consumption and scalability. Consequently, 1-transistor-n-capacitor (1T-nC) ferroelectric random-access memory (FeRAM) has emerged as a compelling non-volatile candidate; however, process-induced variations substantially degrade its operational reliability. This study investigates the impact of wet etch-induced capacitor length (*L*_cap_) variations and atomic layer deposition (ALD)-induced electrode work function (WF) deviations on the sensing margin of 1T-nC FeRAM. The analysis employs Sentaurus TCAD (Synopsys, Inc., Mountain View, CA, USA, Version T-2022.03) simulations calibrated via the Preisach model, utilizing empirical positive-up-negative-down (PUND) measurements of 7 nm Hf_0.5_Zr_0.5_O_2_ (HZO) capacitors. The results demonstrate that geometric shadowing during wet etching induces non-uniform *L*_cap_ profiles. Configuring the bottommost capacitor *L*_cap_ to 80 nm secures a sensing margin exceeding the 150 mV DDR4 specification. Furthermore, TiN oxidation during ALD shifts the plate line work function (WF_PL_). Constraining WF_PL_ between 4.51 eV and 4.71 eV at *L*_cap_ = 90 nm ensures stable read operations, with this window narrowing further as *L*_cap_ is scaled down. By establishing these theoretical boundary conditions, this study provides predictive design guidelines for high-density architectures. Ultimately, mitigating *L*_cap_ geometric dispersion and suppressing TiN oxidation are imperative for guaranteeing sufficient sensing margins, thereby advancing scalable, high-density 1T-nC FeRAM solutions for next-generation AI workloads.

## 1. Introduction

The rapid proliferation of artificial intelligence (AI) and machine learning (ML) workloads has intensified the demand for high-density, non-volatile memory capable of operating beyond the “memory wall” limitations of conventional Von Neumann architectures [1]. Emerging interconnect protocols such as the compute express link (CXL) facilitate unprecedented memory-capacity expansion and cache coherency [2], yet dynamic random-access memory (DRAM), the traditional mainstay of main memory, struggles to fully capitalize on CXL-based topologies: its inherent volatility necessitates continuous refresh operations that incur prohibitive static power consumption, and the physical scaling of the DRAM storage capacitor are rapidly approaching fundamental limits.

As a robust alternative, ferroelectric random-access memory (FeRAM), incorporating hafnium-based ferroelectrics such as Hf_0.5_Zr_0.5_O_2_ (HZO), has attracted significant academic and industrial interest. In contrast to conventional perovskites, HZO demonstrates superior complementary metal-oxide-semiconductor (CMOS) compatibility, elevated coercive fields, and resilient ferroelectricity even at ultra-thin dimensions (e.g., 7 nm), rendering it highly amenable to 3D integration strategies [3].

To transcend the integration density limitations of standard 1-transistor-1-capacitor (1T-1C) configurations, the 1-transistor-n-capacitor (1T-nC) FeRAM architecture has been conceptualized. This topology integrates a singular access transistor with multiple capacitors in a stacked or lateral array, significantly attenuating the effective bit-cell footprint. However, this high-density shared-line architecture imposes a stringent bias scheme to independently access distinct capacitors without inducing destructive read/write disturbances, inherently diminishing the available sensing margin relative to isolated 1T-1C cells. Furthermore, the intricate 3D integration processes essential for 1T-nC FeRAM—such as high-aspect-ratio wet etching and atomic layer deposition (ALD)—introduce pronounced process-induced variations [4]. During crystallization, the mechanical confinement (tensile stress) and oxygen vacancy regulation provided by the TiN electrodes strongly dictate the free energy landscape of the ultra-thin HZO film, fundamentally stabilizing the ferroelectric orthorhombic phase [5,6]. Consequently, process variations not only alter geometries but also critically impact the intrinsic phase stability and macroscopic switching characteristics.

The two process variations addressed in this study arise from distinct steps of this fabrication flow. First, forming the vertically stacked capacitors requires a selective wet etch to open horizontal cavities within a confined 3-D oxide/metal stack. Because this etch process is fundamentally diffusion-limited, the local etch rate—and hence the resulting capacitor length (*L*_cap_)—is intrinsically layer-dependent, as established for TiN wet etching in SC-1 (NH_4_OH/H_2_O_2_) chemistry and for wet etching within other confined nanoscale geometries such as FinFET spacers [7,8]. Although this diffusion-limited etch-rate trend has not been directly re-verified for the exact 3-D stack geometry used in this work, it is adopted here as a literature-motivated boundary condition for the TCAD simulations. Second, forming the TiN/HZO/TiN capacitor stack by ALD exposes only the bottom (plate-line) TiN electrode to the oxidizing ALD ambient during HZO growth, while the top (storage-node) TiN electrode is deposited afterward and is not subjected to this oxidizing exposure. Cross-sectional TEM combined with depth-profiling analysis has directly confirmed the resulting interfacial TiNO_x_ formation and the consequent oxygen-concentration asymmetry between the two electrodes [9], and first-principles and electrode-engineering studies of TiN further confirm that such oxidation and deposition-history differences measurably shift the electrode work function [10,11]. Similarly, the quantitative electrode work function shift adopted in this study is based on literature first-principles and electrode-engineering studies of oxidized TiN rather than a direct measurement on our own capacitors, and is applied here as a literature-motivated modeling assumption. Despite originating from an identical TiN target, the plate-line and storage-node electrodes can therefore acquire different effective work functions (WF_PL_ and WF_SN_, respectively), which is expected to modulate the read characteristics of the shared storage node. Although both variation mechanisms have been individually reported in the literature, their combined impact on the sensing margin of a shared-node 1T-nC array has not been systematically quantified.

An early proof of concept regarding geometric scaling was presented in our prior conference report [12]. Building significantly upon that foundation, this comprehensive study extends the analysis to encompass material-level thermodynamic variations (ALD-induced electrode work function shifts), rigorous array-level parasitic modeling [13,14,15], sub-coercive disturb kinetics [16], and direct material-level verification of the fabricated capacitor stack by cross-sectional electron microscopy. Employing Sentaurus TCAD simulations calibrated with empirical Preisach parameters, we ascertain the optimal predictive boundary conditions required to satisfy the double data rate 4 (DDR4) sensing margin specification of 150 mV [17]. These insights provide foundational guidelines for designing highly reliable FeRAM architectures tailored for demanding AI and ML workloads.

## 2. Materials and Methods

### 2.1. Device Structure and Design Parameters

The 1T-nC FeRAM architecture investigated in this study was previously proposed by our group as a high-density non-volatile memory structure [18]. To improve gate electrostatic control, suppress short-channel effects, and increase spatial density, a double-gate vertical cell transistor (VCT) is employed as the access device [19]. As illustrated in Figure 1a, the cell consists of a VCT, a storage-node contact (SNC), a common storage node (SN), and multiple vertically stacked ferroelectric capacitors (specifically configured as a 1T-4C architecture in this study). The VCT is connected to the common SN through the SNC, while each capacitor is formed between the common SN electrode and an individually addressable plate line (PL). Figure 1b shows the cross-sectional geometry of an individual capacitor and defines the principal structural parameters: the capacitor length (*L*_cap_), width (*W*_cap_), thickness (*t*_cap_), and ferroelectric-layer thickness (*t*_FE_).

The top and bottom electrodes comprising each ferroelectric capacitor are constructed from titanium nitride (TiN), selected to facilitate the formation of the ferroelectric orthorhombic phase by providing mechanical confinement and stress during thermal annealing, a stress-mediated stabilization mechanism recently corroborated by an in situ microscopy study of the TiN/HZO interface [20]. The ferroelectric switching medium consists of a 7 nm HZO film with a nominal Hf:Zr composition of 1:1, synthesized via ALD to guarantee exemplary conformality across the complex 3D topography. Consistent with the established ALD recipe for Hf_0.5_Zr_0.5_O_2_ (equal-cycle TDMA-Hf/TDMA-Zr super-cycles with an O3 oxidant) [9], the 1:1 Hf:Zr stoichiometry in this study is controlled at the process level by fixing the ALD super-cycle ratio between the Hf and Zr precursor pulses at 1:1, rather than being verified after growth by a separate compositional assay; the reliability of this cycle-ratio-controlled approach for obtaining the intended composition and the corresponding ferroelectric response has previously been confirmed by combined cross-sectional TEM and depth-profiling analysis on capacitors grown with the same precursor chemistry [9]. While dedicated elemental analysis (e.g., XPS/EDS) or cross-sectional TEM elemental mapping was not additionally performed on the specific capacitors reported in this study, the process-level 1:1 Hf:Zr stoichiometry control described above, together with the independently validated cross-sectional TEM and depth-profiling compositional analysis referenced in [9] for capacitors grown with the identical ALD precursor chemistry, provide confidence in the intended composition of the HZO film used here. The foundational structural parameters of the capacitors, delineated in Table 1, align with contemporary memory scaling projections. The key fabrication sequence is schematically illustrated in Figure 2. Following the formation of the oxide/metal multilayer stack and side openings, a selective isotropic wet etch is employed to form horizontal cavities. A conformal TiN/HZO/TiN metal-ferroelectric-metal (MFM) stack is subsequently deposited within the recessed regions, followed by ferroelectric annealing and final metallization to complete the vertically integrated capacitor array.

### 2.2. Ferroelectric Characteristics and Calibration

To rigorously map the physical dynamics of the HZO film into the computational domain, empirical evaluations were performed on physical 7 nm metal-ferroelectric-metal (MFM) test structures. The thickness of the deposited HZO layer and the top and bottom TiN electrodes was verified directly by cross-sectional transmission electron microscopy (TEM) on these fabricated capacitors. Representative cross-sectional TEM images are shown in Figure 3. TEM images acquired at multiple locations across the fabricated capacitor consistently showed a uniform HZO layer thickness of approximately 7 nm, matching the ALD process target used throughout the TCAD model in Section 3, with no measurable point-to-point thickness deviation within the resolution of TEM measurements.

Polarization characteristics were ascertained using a Keysight B1500A analyzer (Keysight Technologies, Santa Rosa, CA, USA) via the positive-up-negative-down (PUND) methodology. As illustrated in Figure 4a, the PUND technique isolates the intrinsic ferroelectric switching current from parasitic capacitive and leakage artifacts, thereby facilitating the pristine extraction of the material’s hysteresis loop. The extracted *P*-*E* hysteresis loop in Figure 4b exhibits clear ferroelectric switching behavior, with well-defined remanent polarization (*P*_r_) and coercive field (*E*_c_).

The deduced macroscopic ferroelectric parameters—namely, saturation polarization (*P*_s_), *P*_r_, and *E*_c_—are codified in Table 2. It is noted that macroscopic *P*-*E* hysteresis characteristics inherently exhibit dependencies on the applied poling waveform due to intrinsic switching kinetics. However, for the primary purpose of our study—evaluating the sensing margin and isolating the electrostatic impacts of process variations (such as *L*_cap_ non-uniformity and work function mismatch) on charge-sharing dynamics—the Preisach model calibrated to standard PUND measurements provides a highly robust and widely accepted methodology for device-level simulations [21]. Furthermore, to ensure high predictive accuracy during 100 ns transient operations, the dynamic switching delay parameter (*τ*_p_) was calibrated to 80 ns based on our previous experimental measurements of 7 nm HZO capacitors [22].

### 2.3. Operational Bias Scheme and Data Disturbance

Reliable addressing within the 1T-nC array dictates independent access to distinct cell capacitors to preclude data degradation in unselected (victim) cells during the state manipulation of a selected (target) cell. During a write cycle addressed to the target capacitor, the nominal *V*_CC_ voltage (1.5 V) is subjected across its terminals to induce polarization reversal. Concurrently, fractional biases must be routed to the plate line (PL) and BL of the contiguous victim cells sharing a node. Specifically, an inhibition bias of 1/3 *V*_CC_ is applied to interdict unintended writes [23]. As illustrated in Figure 5a,b, the corresponding bias configurations are applied for writing ‘0’ and ‘1’, respectively, while suppressing unintended polarization switching in the neighboring victim capacitors. The foundational framework and efficacy of this 3-D n-capacitor-stacked architecture and the 1/3 *V*_CC_ inhibition scheme for high-density non-volatile DRAM applications have been systematically validated in our earlier work [18].

Although this 1/3 *V*_CC_ stimulus remains ostensibly beneath the HZO coercive voltage, it precipitates marginal, non-destructive depolarization. This perturbation transiently modifies the effective remanent states, altering the nominal capacitances (*C*_0_ for state ‘0’ and *C*_1_ for state ‘1’). To evaluate these dynamics, the simulation methodology accurately models the applied pulse schemes and the subsequent charge-sharing mechanism. The applied read and write pulses are configured with a 100 ns width and 10 ns rise/fall times. Unlike conventional DRAM, FeRAM provides an intrinsic retention time exceeding 10 years without power [24], eliminating periodic refresh operations.

However, a critical parameter dictating the read operation is the bit-line parasitic capacitance (*C*_BL_). In highly scaled arrays, up to 90% of *C*_BL_ originates from the BL-to-node contact (NC) proximity. Assuming state-of-the-art low-k BL spacer technologies, this capacitance can be minimized to approximately 31.5 aF per cell [13]. To rigorously evaluate our architecture under a worst-case, ultra-high-density scenario, we modeled a sub-array where 512 word-lines (WLs) are connected to a single BL, translating to a massive 2048-bit capacity per BL in a 1T-4C scheme. Under this aggressive configuration, the aggregated *C*_BL_ equates to 16.1 fF (31.5 aF × 512) [15]. By explicitly setting *C*_BL_ = 16 fF in our simulations, we evaluate the sensing margin against severe capacitive loading. Following the write phase and repeated 1/3 *V*_CC_ disturbance pulses (which physically induce accumulative partial depolarization via reverse domain nucleation [16]), the read operation is executed by floating the BL and applying a read pulse to the PL. This initiates charge-sharing between the polarized ferroelectric capacitance and the dominant parasitic BL capacitance (*C*_BL_), generating a distinct BL voltage (*V*_BL_).

Consequently, the sensing margin is quantitatively defined as the final voltage differential between the two logic states (Δ*V*_BL_ = *V*_BL1_ − *V*_BL0_). The simulated transient waveforms explicitly demonstrate that the inhibited cell—having suffered polarization loss—exhibits a reduced *V*_BL1_ and an elevated *V*_BL0_ compared to the ideal target cell, thereby narrowing the effective sensing margin. Accurately modeling this dynamic capacitive ratio is critical for verifying whether the degraded Δ*V*_BL_ satisfies the requisite sensing threshold, substantiating the necessity for further capacitor and array optimizations.

To ensure reproducibility, the Sentaurus TCAD mixed-mode device/circuit simulations employed Fermi-Dirac carrier statistics, doping-dependent Shockley-Read-Hall recombination, Old Slotboom bandgap narrowing, and a doping- and field-dependent carrier mobility model (PhuMob) combined with high-field velocity saturation. The ferroelectric HZO layer was modeled using the built-in Preisach-type polarization model, calibrated to the PUND-extracted *P*_s_, *P*_r_, and *E*_c_ values (Table 2) and to the *τ*_p_ = 80 ns dynamic switching parameter obtained from our previous 7 nm HZO capacitor measurements [22]. At the circuit level, the peripheral bit-line access transistors were represented by a compact MOSFET model rather than by full 3-D device structures, and all write, inhibit, and read pulses were generated as ideal piecewise-linear voltage sources; besides the intentional *C*_BL_ = 16 fF bit-line loading discussed above, no additional parasitic resistance or capacitance was included in the circuit-level model. The initial DC operating point was established through a staged Newton (Coupled) solve that sequentially activates the Poisson equation, the electrical contacts, the external circuit, and finally the electron and hole continuity equations, after which the transient simulation was advanced with an initial and maximum time step equal to one-twentieth of the pulse rise/fall time (0.5 ns), a minimum step of 1 × 10^−21^ s, relative-error-controlled convergence to 5 significant digits, and the Pardiso direct linear solver.

The complete read/write/inhibit sequence used throughout this study is illustrated schematically in Figure 5a,b for the write ‘0’ and write ‘1’ phases, respectively. During a write ‘0’ operation, the target cell’s plate line (PL) and bit line (BL1) are driven to +*V*_CC_ and 0 V, respectively, while the adjacent victim cell sharing the same node is inhibited by simultaneously biasing its plate line at +1/3 *V*_CC_ and its bit line (BL2) at +2/3 *V*_CC_; during a write ‘1’ operation, these two inhibit levels are exchanged (PL at +2/3 *V*_CC_ and BL2 at +1/3 *V*_CC_), while the target cell’s PL and BL1 are set to 0 V and +*V*_CC_, respectively. Because the net voltage actually developed across each inhibited (victim) capacitor equals the difference between its two applied inhibit levels, this complementary biasing scheme yields the same effective disturb voltage, 1/3 *V*_CC_, on the victim capacitor in both the write ‘0’ and write ‘1’ phases, consistent with the inhibition scheme validated in our earlier work [18]. To emulate realistic array-level disturb accumulation rather than a single isolated pulse, each victim capacitor was subjected to 10 consecutive write/inhibit cycles (100 ns pulse width, 10 ns rise/fall time, separated by a 100 ns inter-pulse hold interval) prior to the final read-margin evaluation, consistent with the repeated 1/3 *V*_CC_ inhibit-endurance behavior reported for the same inhibition scheme in our earlier work [18].

## 3. Results

The results presented below combine two distinct types of evidence. Section 2.2 and the electron-microscopy analysis above are empirical, obtained directly from the fabricated 7 nm HZO MFM capacitors (cross-sectional TEM, PUND hysteresis measurement, and dynamic polarization measurement). All sensing margin results presented in this section (Table 3 and Table 4, and Figure 6, Figure 7 and Figure 8) are computational, obtained from Sentaurus TCAD simulations that are calibrated to, but distinct from, these empirical inputs.

### 3.1. Impact of Wet Etch-Induced L_cap_ Variation

The realization of the 1T-nC FeRAM matrix necessitates sophisticated, high-aspect-ratio subtractive patterning. A highly selective wet etching protocol is predominantly utilized for sacrificial layer excision and TiN electrode definition. Wet etching is fundamentally predicated on isotropic chemical dissolution; however, it intrinsically exhibits localized etch-rate perturbations dictated by topographical constraints. As illustrated in Figure 6a, the wet etch rate is higher in the upper layers and gradually decreases toward the lower layers of the stacked structure. The resulting layer-dependent variation in *L*_cap_ is schematically shown in Figure 6b.

This layer-dependent variation in *L*_cap_ is an inevitable consequence of the diffusion-limited etch kinetics inherent in high-aspect-ratio 3D stacked structures [7,8]. As the etchant penetrates deeper into the vertical stack, mass transport (diffusion) becomes highly restricted, leading to slower lateral etch rates at the bottommost layers. Consequently, a pronounced layer-dependent *L*_cap_ profile emerges, with the bottommost capacitors undergoing insufficient lateral etching and therefore exhibiting the minimum *L*_cap_ within the stacked structure.

To quantitatively evaluate the ramifications of this spatial variance on array reliability, *L*_cap_ was computationally modulated from 60 nm to 110 nm (10 nm resolution), and the sensing margin was extracted across all theoretical target–victim permutations.

Table 3 comprehensively catalogues the extracted sensing margins across all theoretical target–victim *L*_cap_ permutations. A pronounced electrical asymmetry manifests depending on the target–victim assignment, revealing that the sensing margin is dominantly dictated by the victim cell’s dimension. For example, a target element of *L*_cap_ = 80 nm subjected to inhibition from a victim element of *L*_cap_ = 90 nm yields a robust margin of 172.2 mV. Conversely, reciprocating the topology (target *L*_cap_ = 90 nm, victim *L*_cap_ = 80 nm) degrades the margin to 154.3 mV. More broadly, the full matrix elucidates a distinct overarching trend: expanding the victim *L*_cap_ significantly enhances the read margin (transitioning from the ≈115 mV regime to the ≈205 mV regime), whereas variations in the target *L*_cap_ introduce merely marginal perturbations, typically yielding fluctuations of less than 5 mV within a constant victim column. This pronounced dependency on the victim dimension is intrinsically linked to the charge-sharing dynamics at the common storage node. Because the stacked capacitors operate in parallel at this shared node, an enlarged victim area proportionally increases the aggregate cell capacitance; this favorable modulates the voltage division ratio against the parasitic *C*_BL_, thereby amplifying Δ*V*_BL_ during the read operation.

To translate these asymmetric interactions into a practical array-level design rule, Figure 7 re-evaluates the sensing margin distribution as a function of the minimum dimensional constraint, *L*_cap,min_, for any given target–victim pair. While the upper boundary of the shaded envelope reflects highly favorable conditions driven by oversized victim cells, system-wide read reliability is strictly dictated by the worst-case scenarios, which are represented by the lower bound. As illustrated, aggressive scaling that allows *L*_cap,min_ to drop below 80 nm forces these worst-case margins to violate the 150 mV threshold required by the DDR4 standard. Therefore, securing a minimum footprint of 80 nm is strongly recommended as a practical design rule under the *C*_BL_, *V*_CC_, and HZO material parameters considered in this study. In the context of the 3D stacked architecture and its inherent wet etch shadowing effects, this establishes a practical process guideline: if the bottommost (and thus most restricted) capacitor is patterned to a minimum of 80 nm, the simulations indicate that all superjacent layers (*L*_cap_ ≥ 80 nm) are expected to meet the operational tolerance under the modeled conditions. Because *L*_cap_ was swept in 10 nm increments, 80 nm represents the first simulated grid point at which the worst-case margin satisfies the 150 mV criterion, rather than a continuously resolved physical threshold; a finer-resolution sweep would be required to pinpoint the exact boundary more precisely.

### 3.2. Impact of Electrode Work Function Variation

In conjunction with structural aberrations, the sequential thermodynamic processing inherent in 1T-nC fabrication introduces severe material incompatibility. Specifically, while identical TiN precursors define both the PL and SN interfaces, their resultant effective work functions (WF) frequently diverge.

This asymmetrical divergence is governed by disparate thermal and oxidative exposure. During the ALD of the 7 nm HZO dielectric at elevated thermodynamic states (typically ≈250 °C in oxidizing ambient), the antecedent PL TiN electrode undergoes parasitic interfacial oxidation, forming an interfacial TiNO_x_ layer [9]. The resultant TiNO_x_ layer structurally modulates the effective PL work function (WF_PL_); first-principles calculations of oxidized TiN surfaces predict an increase in work function relative to the stoichiometric surface upon oxidation, with the magnitude depending on the exposed crystallographic texture (e.g., an increase of approximately 0.75 eV for the technologically dominant TiN(111) orientation) [10], and electrode-engineering studies of TiN top electrodes in HZO capacitors have likewise confirmed that deposition history and oxidation state measurably alter both electrode composition and device characteristics [11], with a recent hybrid TiN/W electrode study further demonstrating that inserting a bottom-side metal buffer layer suppresses this interfacial oxidation while simultaneously enhancing the stress-stabilized ferroelectric response [25]. Conversely, the superjacent SN electrode (WF_SN_), deposited post-ALD, circumvents this specific oxidizing phase, preserving its elemental WF properties. It is noted that the approximately 0.75 eV work function shift and its underlying trend are adopted from literature first-principles and electrode-engineering studies [10,11], rather than independently re-derived or measured for the exact TiN/HZO stack used in this work, and are applied here as a literature-motivated modeling assumption to bound the plate-line work function window.

To rigorously evaluate the electrical impact of this phenomenon, WF_SN_ was anchored at an idealized 4.66 eV, and WF_PL_ was computationally swept from 4.40 eV to 4.90 eV with high-resolution increments (0.01 eV). *L*_cap_ was held constant at 90 nm to isolate work function deviations from geometric effects. Table 4 indicates that achieving a sensing margin more than 150 mV requires strict confinement of WF_PL_ to a highly precise window between 4.51 eV and 4.71 eV.

Figure 8a summarizes the allowable WF_PL_ ranges that satisfy the 150 mV sensing margin criterion for different *L*_cap_ values. The acceptable WF_PL_ window narrows as *L*_cap_ decreases: 4.59–4.70 eV for *L*_cap_ = 80 nm, 4.51–4.71 eV for *L*_cap_ = 90 nm, and 4.47–4.80 eV for *L*_cap_ = 100 nm. These results indicate that capacitor scaling increases the sensitivity of the sensing margin to WF_PL_ variation. For all investigated *L*_cap_ values, the maximum sensing margin is obtained when WF_PL_ approaches WF_SN_, demonstrating the importance of minimizing the electrode work function difference.

The underlying physical mechanism driving this degradation is elucidated in the *P*-*V* hysteresis dynamics as depicted in Figure 8b. The asymmetric work functions induce a static internal electric field across the ferroelectric volume, manifesting as a rigid, parallel shift in the hysteresis loop along the voltage axis. While mitigating protocols exist for singular 1T-1C nodes, within the multi-capacitor 1T-nC environment, the rigid 1/3 *V*_CC_ inhibit bias falls into deterministic misalignment with the shifted coercive potentials. Should this hysteresis shift exceed nominal margins, the inhibit bias inadvertently provokes severe dynamic degradation of *C*_1_, exponentially compounding read failure.

## 4. Discussion

The empirical and computational observations derived from this study underscore the extreme susceptibility of 1T-nC FeRAM configurations to process-induced non-idealities. Unlike conventional 1T-1C architectures, where process variations primarily affect isolated cells, the 3D topology of the 1T-nC structure tightly couples multiple capacitors to a single storage node. Consequently, spatial geometric drift (e.g., wet etch shadowing) and sequential thermodynamic variance (e.g., ALD budget asymmetry) are not only aggregated but inherently amplified within the shared node. As the number of vertically stacked capacitors increases to boost areal density, this cumulative variance becomes increasingly fatal, severely degrading the uniformity of the sensing margins across the array.

Imposing the 80 nm *L*_cap_ boundary constraint is strongly recommended for preserving read reliability using contemporary wet etch chemistries; the assumption of such layer-dependent profiles is fundamentally grounded in diffusion-limited etch kinetics inherent in confined, high-aspect-ratio lateral recesses. Aggressive dimensional shrinkage beyond this threshold fundamentally necessitates disruptive innovations in isotropic etching technologies. However, it is imperative to acknowledge that this 80 nm criterion is strictly contingent upon the specific parameters established in this study—namely, *C*_BL_, *V*_CC_, and the baseline HZO ferroelectric characteristics. For instance, if *C*_BL_ expands due to extensive array scaling, or if *V*_CC_ is reduced to accommodate low-power applications, the vulnerability to these structural variations will inevitably worsen, thereby drastically shrinking the viable process window. Conversely, if future advancements in material engineering yield enhanced HZO characteristics—such as a significantly higher *P*_r_—this variation-induced degradation can be substantially alleviated, potentially relaxing the rigid *L*_cap_ constraint.

Recent advances in predictive dynamic-switching models, which link time-varying domain-wall kinetics directly to material- and circuit-level design parameters, offer a promising route toward systematically identifying such HZO improvements [26].

In addition to the lateral *L*_cap_ variation analyzed above, a vertical ferroelectric-thickness (*t*_FE_) variation could, in principle, also perturb the sensing margin: because the ferroelectric capacitance scales approximately as *C*_FE_ proportional to 1/*t*_FE_ and the coercive voltage scales approximately with *E*_c_ times *t*_FE_, a local reduction in HZO thickness would increase the cell capacitance and lower the switching voltage, perturbing the charge-sharing ratio against *C*_BL_ in a qualitatively similar direction to an *L*_cap_ increase. However, as reported in Section 2.2, cross-sectional TEM performed at multiple locations across our fabricated capacitors showed no measurable point-to-point HZO thickness deviation, with all locations consistently confirming the ≈7 nm process target. We therefore conclude that, for the process flow considered in this study, *L*_cap_ and WF_PL_ variation are the dominant sources of sensing margin degradation, while *t*_FE_ dispersion is not a significant contributor.

Furthermore, the degradation of the sensing margin directly impacts the temporal performance of the memory array. In conventional latch-based CMOS sense amplifiers, the resolution delay (*t*_sense_) follows a logarithmic relationship with the initial voltage differential (*t*_sense_ is proportional to ln(*V*_swing_/Δ*V*_BL_)), where *V*_swing_ denotes the target output voltage swing of the sense amplifier (typically the full logic level, *V*_CC_) [27]. Therefore, a reduced Δ*V*_BL_, induced by dimensional shrinkage, work function mismatch, or accumulative sub-coercive disturb, forces the sense amplifier to expend substantially more time to resolve the bit state, severely increasing the sensing delay and degrading the overall system bandwidth.

Concurrently, minimizing the structural oxidation of the TiN plate line constitutes a critical integration mandate. To arrest the parasitic hysteresis shift and secure the operational WF_PL_ window (4.51 eV to 4.71 eV for a 90 nm structure), advanced metallurgical engineering is imperative. The post-deposition integration of high-pressure annealing (HPA) presents a scientifically sound stratagem to effectively suppress interstitial TiNO_x_ defect propagation, passivate interface states, and recalibrate the targeted work function [28].

Overall, these results demonstrate that reliable operation of the vertically stacked 1T-nC FeRAM architecture requires simultaneous control of capacitor geometry and electrode work function. While independent deterministic sweeps cannot establish complete statistical yield, the *L*_cap_ and WF_PL_/SN process windows identified in this study provide critical theoretical boundaries and predictive design guidelines for satisfying the sensing margin requirement appropriate to the target technology and memory architecture.

## 5. Conclusions

This study established two quantitative process variation boundaries for reliable sensing in high-density 1T-nC FeRAM, derived from TCAD simulations calibrated against fabricated 7 nm HZO capacitors. First, layer-dependent wet etch shadowing requires the bottommost (minimum) capacitor length, *L*_cap,min_, to be maintained at 80 nm or greater; below this value, the worst-case target–victim sensing margin falls below the 150 mV DDR4 specification, whereas *L*_cap,min_ ≥ 80 nm is expected to satisfy Δ*V*_BL_ > 150 mV across all evaluated target–victim combinations under the conditions modeled in this study (Table 3, Figure 7). Second, ALD-induced oxidation of the plate-line TiN electrode requires the plate-line work function (WF_PL_) to remain within a bounded window around the storage-node work function (WF_SN_ = 4.66 eV): 4.59–4.70 eV at *L*_cap_ = 80 nm, 4.51–4.71 eV at *L*_cap_ = 90 nm, and 4.47–4.80 eV at *L*_cap_ = 100 nm (Table 4, Figure 8a), within the parameter space evaluated in this study; this window narrows as *L*_cap_ is scaled down, indicating that smaller cells are increasingly sensitive to electrode work function asymmetry. Cross-sectional TEM confirmed a uniform ≈ 7 nm HZO thickness across the fabricated capacitor with no measurable point-to-point deviation, indicating that, under the process flow considered here, *L*_cap_ and WF_PL_ variation—rather than ferroelectric thickness variation—are the dominant sources of sensing margin degradation. Because the sensing delay of a latch-based CMOS sense amplifier scales logarithmically with 1/Δ*V*_BL_, the same *L*_cap_ and WF_PL_ boundaries that protect the sensing margin also bound the worst-case sensing delay. Taken together, these *L*_cap_ and WF_PL_/SN boundary conditions constitute predictive design guidelines for the geometric and electrode-material control required to sustain sensing margin reliability in scaled, shared-node 1T-nC FeRAM arrays.

## Figures and Tables

**Figure 1:**
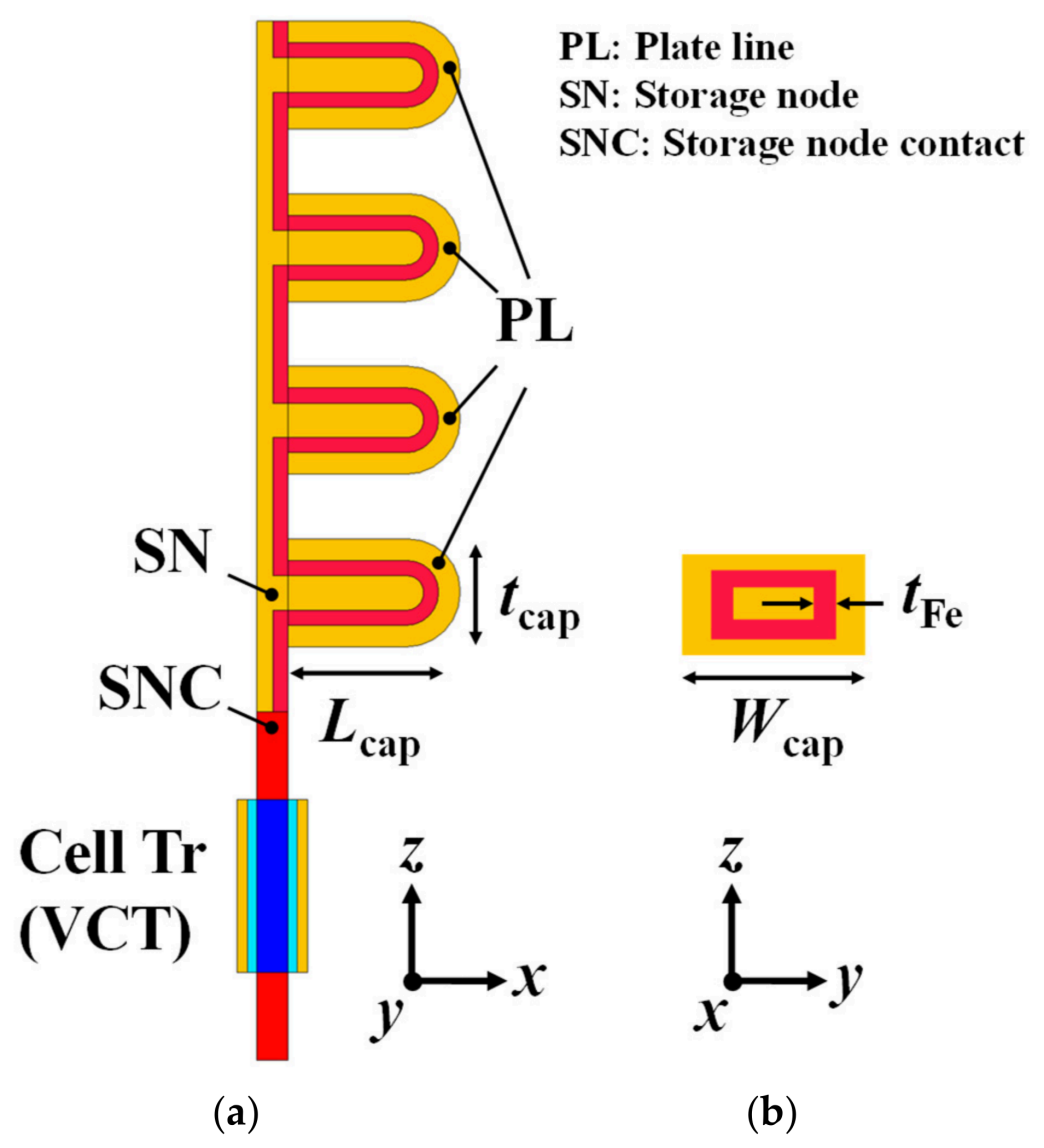
Device structure of 1T-nC FeRAM (cut view of (**a**) x-z plane and (**b**) y-z plane).

**Figure 2:**
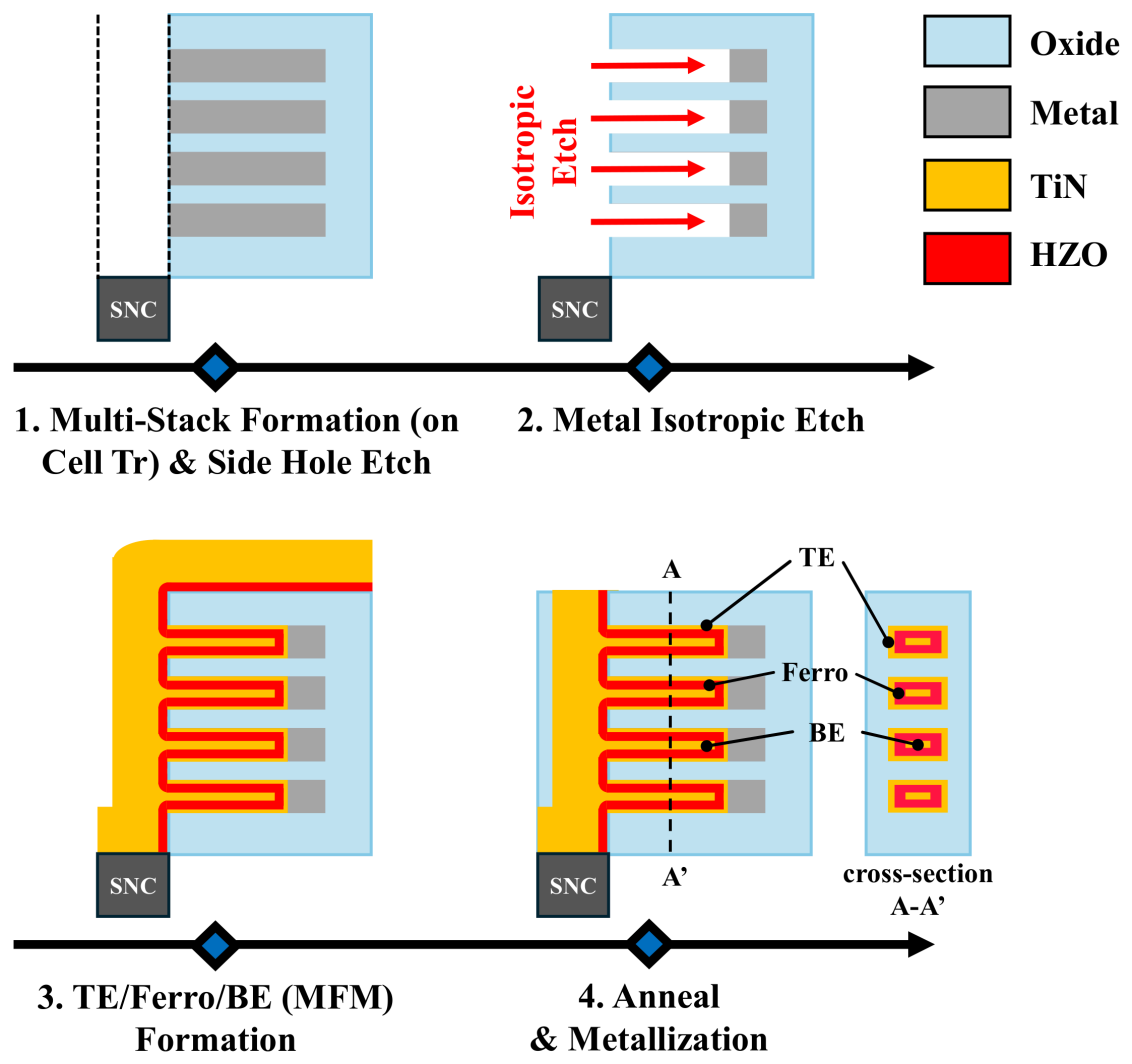
Key fabrication process flow of the n-stacked capacitor structure.

**Figure 3:**
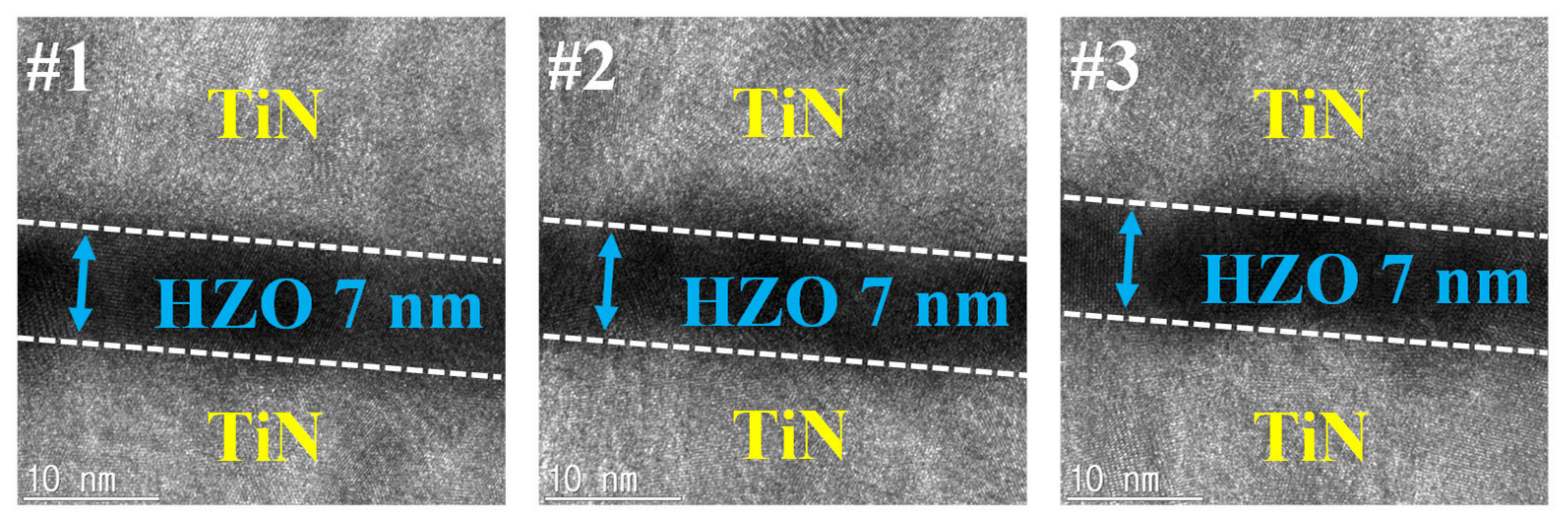
Representative cross-sectional transmission electron microscopy (TEM) images of the fabricated TiN/HZO/TiN metal-ferroelectric-metal (MFM) capacitor, acquired at three different locations along the same capacitor, confirm a uniform ≈ 7 nm HZO film thickness between the top and bottom TiN electrodes at every location.

**Figure 4:**
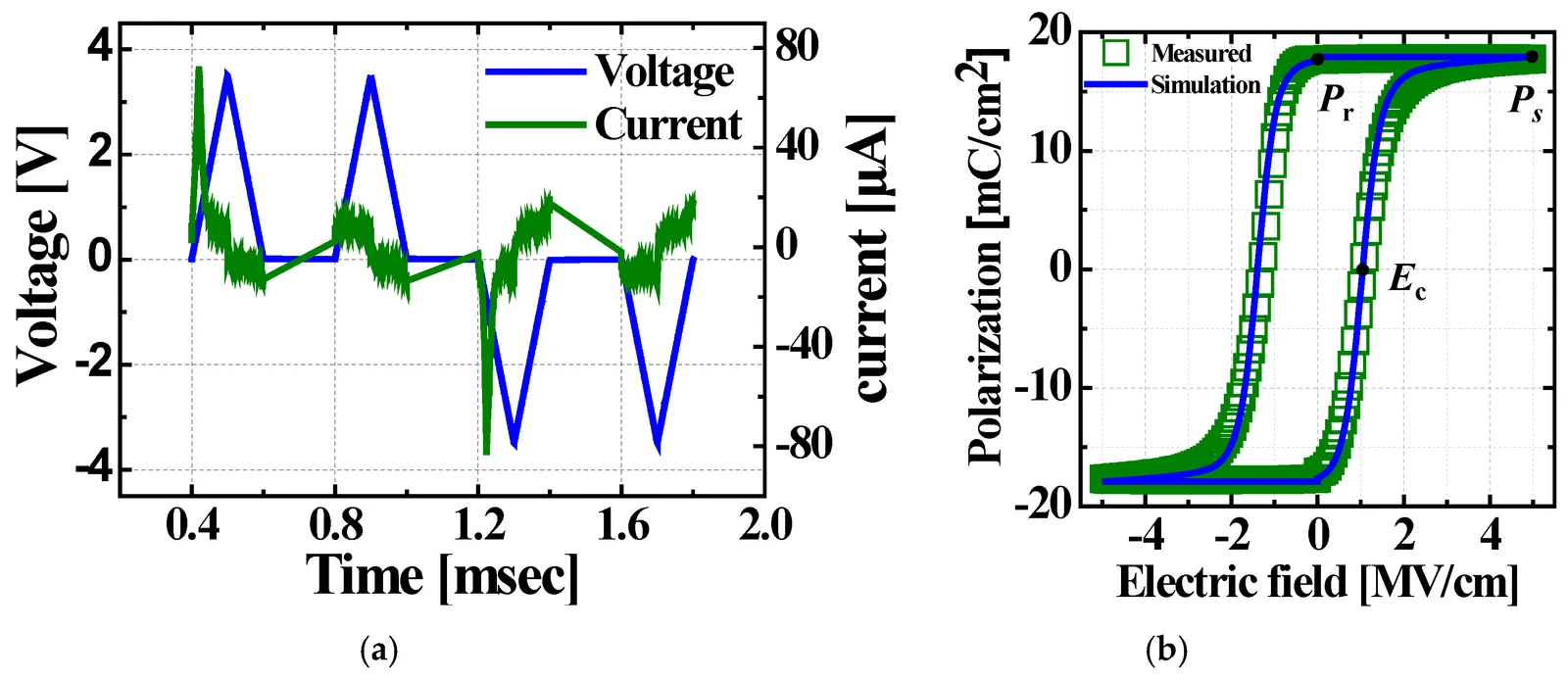
(**a**) PUND measurement waveform of fabricated 7 nm MFM capacitor and (**b**) extracted *P*-*E* hysteresis loop fitted by TCAD using the Preisach model.

**Figure 5:**
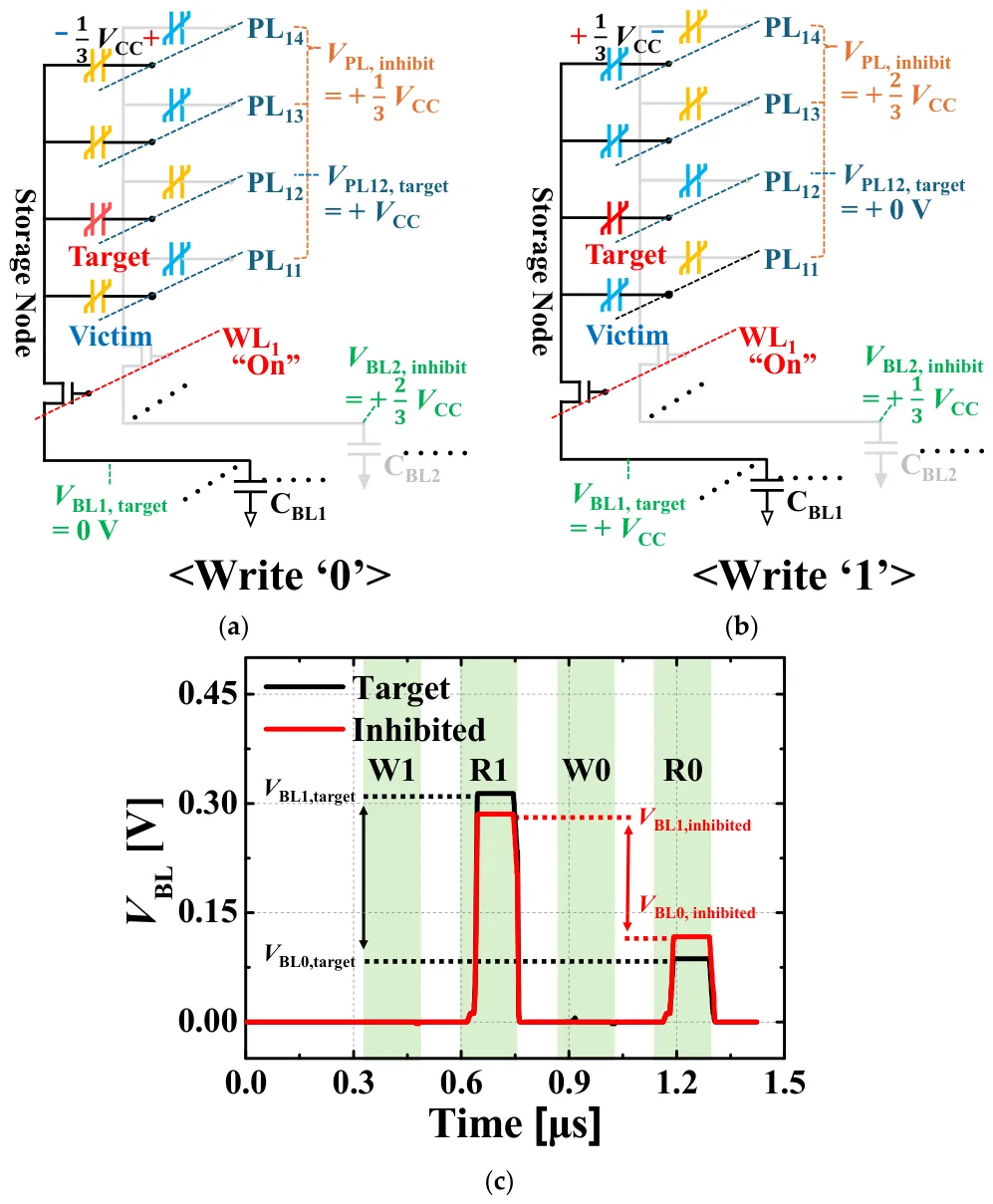
(**a**) 1T-nC bias scheme during write ‘0’ and (**b**) write ‘1’, and (**c**) transient bit line voltage waveforms demonstrating sensing margin degradation in the inhibited cell. In (**a**) and (**b**), the red capacitor symbol denotes the target cell, while blue and yellow symbols denote victim cells biased at −1/3 *V*_CC_ and +1/3 *V*_CC_, respectively. The read and write operations were simulated using a pulse width of 100 ns with 10 ns rise and fall times to reflect high-speed memory operation.

**Figure 6:**
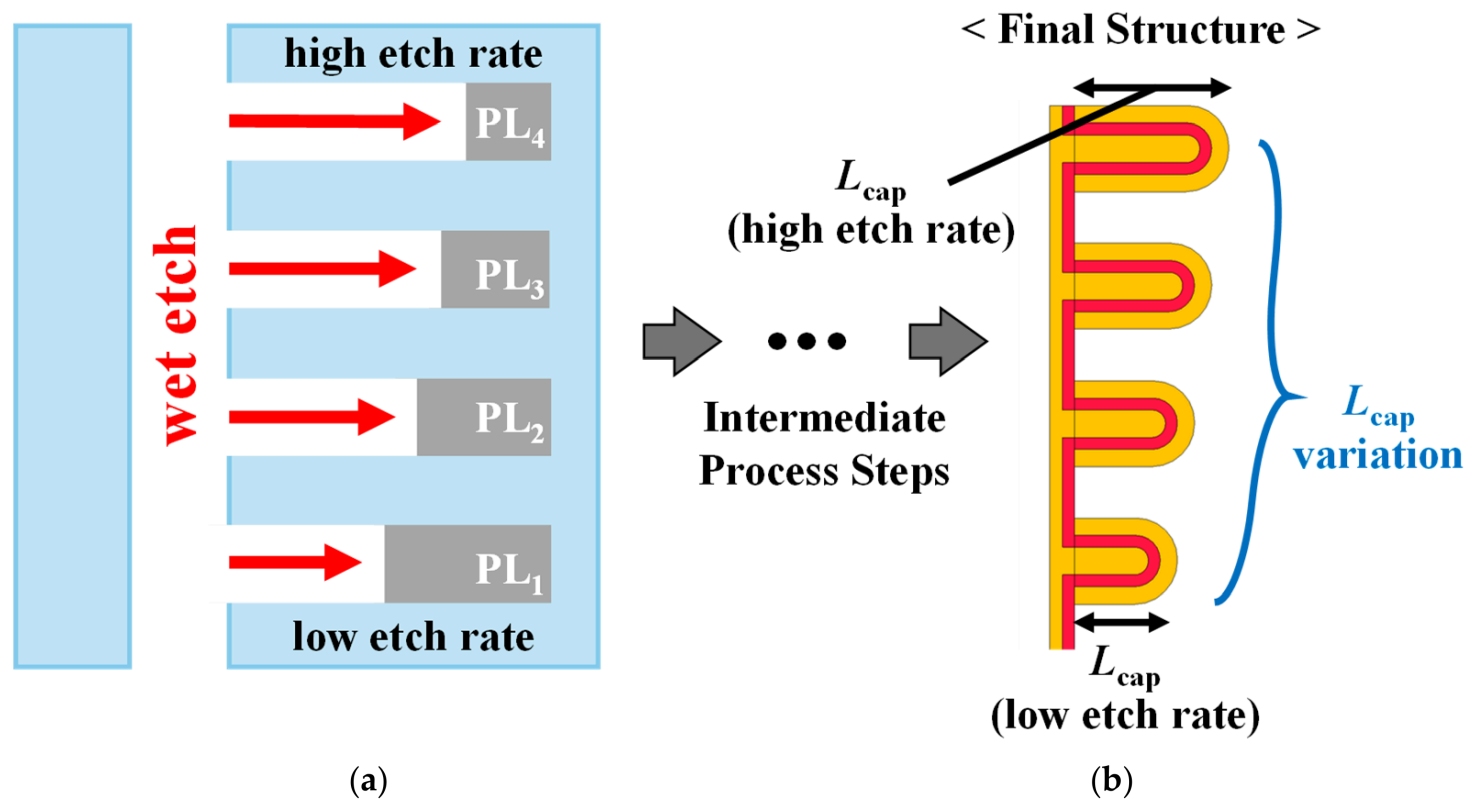
(**a**) TiN wet etching process and layer-dependent etch rate non-uniformity. (**b**) 1T-nC FeRAM capacitors with variation in *L*_cap_.

**Figure 7:**
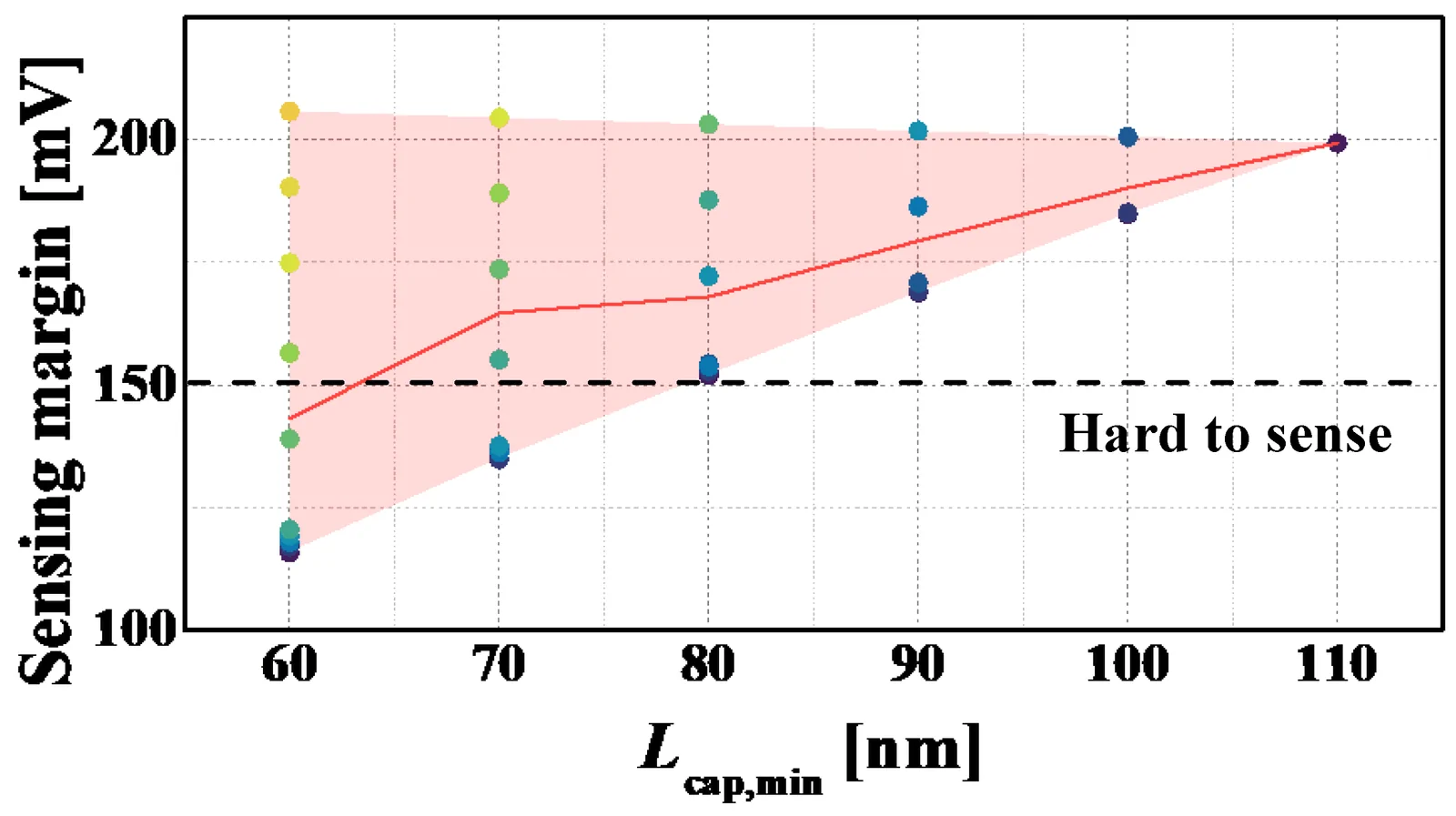
Distribution of sensing margins as a function of the minimum *L*_cap_ within the target–victim combinations. The lower bound of the envelope dictates the worst-case margin, establishing *L*_cap,min_ as a critical process criterion to avoid the “hard to sense” regime.

**Figure 8:**
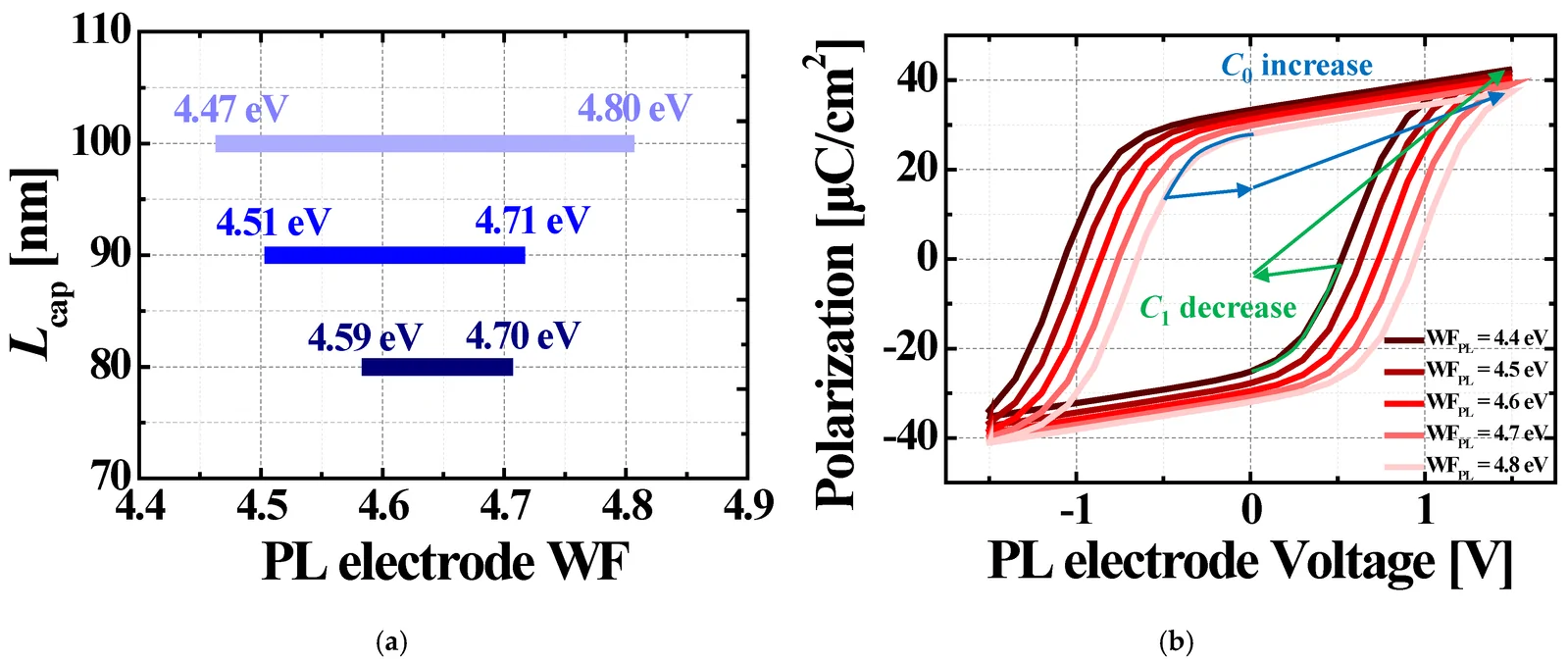
(**a**) The allowable range of the plate line electrode work function varies with *L*_cap_. (**b**) The hysteresis curve of the *P*-*V* characteristic shifts in parallel according to the change in the work function of the plate line electrode.

**Table 1:** FeRAM design parameters.

Parameter	Value
Capacitor length (*L*_cap_)	90 nm
Capacitor width (*W*_cap_)	60 nm
Capacitor thickness (*t*_cap_)	50 nm
Effective capacitor area (*A*_cap_)	1.24 × 10^−2^ μm^2^
Ferroelectric thickness (*t*_Fe_)	7 nm

**Table 2:** Extracted ferroelectric parameters (7 nm HZO).

Parameter	Value
Saturation Polarization (*P*_s_)	17.8 μC/cm^2^
Remanent Polarization (*P*_r_)	17.7 μC/cm^2^
Coercive Field (*E*_c_)	1.2 MV/cm

**Table 3:** Sensing margins for different target- and victim-cell *L*_cap_ combinations (in mV). Bold values indicate sensing margins that meet or exceed the DDR4 specification of 150 mV.

Target *L*_cap_	Victim *L*_cap_					
	60 nm	70 nm	80 nm	90 nm	100 nm	110 nm
60 nm	120.6	139.0	**156.5**	**174.9**	**190.4**	**205.8**
70 nm	119.3	137.6	**155.2**	**173.6**	**189.1**	**204.5**
80 nm	118.0	136.3	**153.8**	**172.2**	**187.7**	**203.2**
90 nm	118.0	136.6	**154.3**	**170.9**	**186.4**	**201.8**
100 nm	117.1	134.9	**152.9**	**169.1**	**185.1**	**200.6**
110 nm	115.8	134.9	**152.0**	**168.8**	**184.8**	**199.3**

**Table 4:** Sensing margin for different target- and victim-cell plate line electrode work function (WF_PL_) combinations (in mV). Bold values indicate sensing margins that meet or exceed the DDR4 specification of 150 mV.

Target WF	Victim WF							
	4.40 eV	4.50 eV	4.51 eV	4.55 eV	4.60 eV	4.66 eV	4.71 eV	4.80 eV
4.40 eV	106.0	134.9	136.9	143.2	146.8	144.8	139.7	114.6
4.50 eV	119.8	148.6	**150.6**	**156.7**	**160.1**	**157.7**	**152.3**	126.1
4.51 eV	121.0	149.7	**151.7**	**157.9**	**161.2**	**158.8**	**153.3**	127.0
4.55 eV	124.9	**153.9**	**155.9**	**162.0**	**165.3**	**162.7**	**157.1**	130.4
4.60 eV	129.8	**158.3**	**160.3**	**166.3**	**169.5**	**166.7**	**161.0**	133.9
4.66 eV	133.9	**162.3**	**164.3**	**170.2**	**173.3**	**170.5**	**164.6**	137.1
4.71 eV	136.0	**164.4**	**166.3**	**172.3**	**175.3**	**172.4**	**166.5**	138.7
4.80 eV	139.6	**167.9**	**169.8**	**175.7**	**178.7**	**175.7**	**169.6**	141.5

## Data Availability

The data presented in this study are available upon reasonable request to the corresponding author.
